# Care Management and Survival of Patients Diagnosed with Synchronous Metastatic Colorectal Cancer: A High-Resolution Population-Based Study in Two French Areas

**DOI:** 10.3390/cancers14071777

**Published:** 2022-03-31

**Authors:** Andrea Mulliri, Joséphine Gardy, Mélanie Cariou, Guy Launoy, Michel Robaszkiewicz, Arnaud Alves, Véronique Bouvier, Olivier Dejardin

**Affiliations:** 1 ANTICIPE U1086 INSERM-UCN, Centre François Baclesse, 14000 Caen, France; josephine.gardy@inserm.fr (J.G.); guy.launoy@unicaen.fr (G.L.); alves-a@chu-caen.fr (A.A.); veronique.bouvier@unicaen.fr (V.B.); olivier.dejardin@unicaen.fr (O.D.); 2 Department of Digestive Surgery, University Hospital of Caen, Avenue de la Côte de Nacre, CEDEX, 14033 Caen, France; 3 Calvados Digestive Cancer Registry, University Hospital of Caen, 14000 Caen, France; 4 Finistere Digestive Cancer Registry, University Hospital of Brest, CEDEX, 29200 Brest, France; melanie.cariou@chu-brest.fr (M.C.); michel.robaszkiewicz@chu-brest.fr (M.R.); 5 Epidemiology Research and Evaluation Unit, Department of Research, University Hospital of Caen, Avenue de la Côte de Nacre, CEDEX, 14033 Caen, France

**Keywords:** digestive cancer, stage IV, prognosis, registry

## Abstract

**Simple Summary:**

The management of colorectal cancer with synchronous metastasis is complex and heterogenous depending on numerous factors related to patients and health care organisation. The description of real-word medical practices is challenging. While hospital series are mainly promoted by highly specialised hospitals and are not in position to accurately measure the heterogeneity of patients and practices, cancer registries are essential to describe such practices in the area covered with no regards to hospital status nor degree of specialisation. The goals of our study were to describe the colorectal cancer with synchronous metastasis population and to evaluate the impact of the different treatments on survival. Our results indicate that a combined treatment, chemotherapy and primary tumor resection, is the cornerstone of oncological management, with survival being negatively impacted by other treatment strategies.

**Abstract:**

Population-based studies provide the opportunity to assess the real-world applicability of current clinical practices. The present research evaluated the survival outcomes of different therapeutic strategies for colorectal cancer (CRC) with synchronous metastasis (SM). The differential impact of treatment sequence, viz. whether chemotherapy (CT) or primary tumor resection (PTR) was performed first, was also evaluated. Methods: All CRC cases with SM diagnosed between 2006 and 2016 (*N* = 3062) were selected from two specialized digestive cancer registries from northwest France. Cox regression analysis was used to assess survival. Multivariable logistic regression was used to examine factors related to the combination of PTR and CT. Results: The longest survival was observed in patients treated by PTR combined with CT (Group 4; *N* = 1159). Overall survival was 51.80% at one year (95% Confidence Interval (CI) 50.00–53.60%) and 9.40% at five years (95% CI, 8.30–10.60%). Survival did not differ with respect to the order of treatment in multivariable analysis (hazard ratio, 1.05; 95% CI, 0.88–1.24; *p* = 0.55). Conclusion: Regardless of the sequence of treatment, a PTR + CT offered the best survival in patients with CRC and SM, even though few were eligible for combination therapy (38%).

## 1. Introduction

The number of colorectal cancer (CRC) cases diagnosed with synchronous metastasis (SM) at the time of diagnosis is increasing [1]. However, the definition of SM remains an issue of debate [2]. CRC with SM is usually considered if metastases are present at diagnosis or within 6 months of initial diagnosis. Approximately 20–25% of patients with CRC have SM [3,4]. Historically, patients with CRC and SM were considered potential candidates for primary tumor resection (PTR) to prevent local complications [5]. However, the indication for systematic PTR in patients with SM is still debated [4,6], although the therapeutic strategy must be discussed on a case-to-case basis. Currently, chemotherapy (CT) is the first-line and most frequently performed treatment for noncomplicated CRC with SM [4,6]. Previous attempts to conduct randomized controlled trials with patients with CRC and SM have prematurely been stopped due to poor recruitment. A phase III randomized controlled trial has just been published comparing the outcomes of PTR treatment versus CT for CRC and SM. This study was stopped prematurely due to futility [7]. A number of other trials are, however, ongoing [8,9]. In addition to randomized controlled trials, population-based studies are essential to assess the real-world applicability of current clinical practices.

The main goal of our study was to evaluate the impact of CT and PTR on survival in patients with CRC and SM. We also evaluated the factors associated with treatment outcomes, as well as the differential impact of treatment order sequence (CT vs. PTR) on therapeutic outcomes.

## 2. Materials and Methods

### 2.1. Population

**Figure 1:**
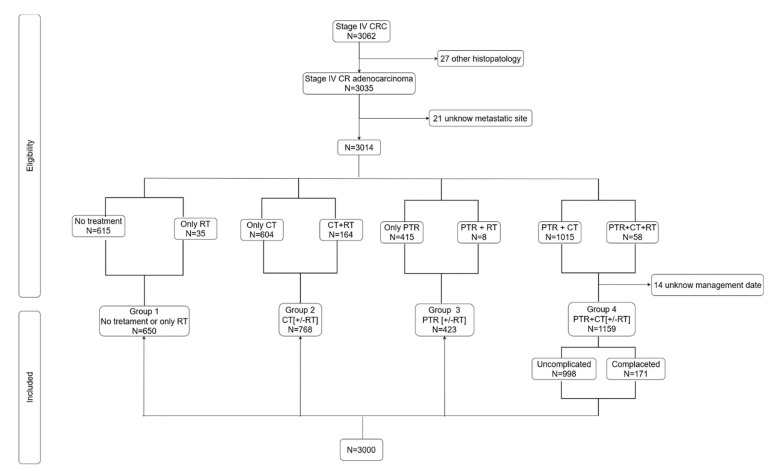
Flowchart. All patients with colorectal cancer (CRC) and synchronous metastases (SM) diagnosed at two specialized digestive cancer registries (*N* = 3062).

All patients diagnosed with CRC and SM (i.e., metastatic at diagnosis or appearance of metastases within 6 months of diagnosis), between 2006 and 2016, from the Finistere and Calvados digestive cancer registries (https://lesdonnees.e-cancer.fr/Informations/Sources/SOURCE-Reseau-FRANCIM; accessed on 30 March 2022) (*N* = 3062; Figure 1) were eligible for enrolment.

The resident population of these two well-defined administrative areas (Calvados and Finistère department) included 1,601,928 inhabitants in 2016. Both digestive cancers use identical standardized data collection, recording, and validation procedures. These two databases are registered with the National Commission on Information Technology and Civil Liberties (CNIL), and the quality of the data collected is evaluated every 4 years by the Institut National de la Santé et de la Recherche Médicale (INSERM), “Santé Publique France,” and the Institut National du Cancer (INCa).

In 99.12% (*N* = 3035) of cases, a colorectal adenocarcinoma was recorded. CRC with a different histology than adenocarcinoma (*N* = 27) and patients for whom the metastatic location was unknown were excluded (*N* = 21).

The patients with CRC and SM were divided into four intention-to-treat groups based on the treatment received: Group 1, no treatment or only radiotherapy (RT); Group 2, CT (+/− RT); Group 3, PTR (+/− RT); and Group 4, combination of PTR and CT (+/− RT). The PTR group included all patients with oncological colorectal resection (i.e., colectomy or proctectomy). All surgical derivations (including bypass stoma or surgical internal bypass) in the first instance before PTR were considered as PTR. In contrast, patients with only bypass (*N* = 21) or stomy (*N* = 117) without colorectal tumor resection were not included in the PTR group.

In Group 4, 14 patients were excluded, as the date of treatment was not known. The final analysis was based on the data of 3000 patients.

To study the effect of treatment order sequence (CT vs. PTR) on survival outcomes, a subgroup analysis was performed, with 22.4% (*N* = 260) of patients having undergone CT first, and the other 77.6% (*N* = 899) having received PTR first.

### 2.2. Covariates

The following data were routinely collected: sex, age, date of diagnosis, histopathology and site of the primary tumor, localization of SM, and presentation at a multidisciplinary consultation. For analysis, age at diagnosis was classified into the following four age groups: <60 years, 60–69 years, 70–79 years, and ≥80 years. The year of diagnosis was divided into the following three groups for analysis: 2006–2008, 2009–2012, and 2013–2016. Tumor histopathology was classified according to the International Classification of Disease for Oncology (ICD-O-3). Comorbidities were assessed using the Charlson comorbidity index (CCI) [10]. The CCI was grouped for analysis: 0, 1, 2, or >2. The localization of the primary tumor was classified as “colon” or “rectum”. Tumors with a rectosigmoid localization were included in the “colon” localization group (*N* = 711; 23.92%).

The localization of SM was divided into four groups for analysis: intra-abdominal (peritoneum, localized organ, or intra-abdominal lymph nodes; 69.11%); intrathoracic (lung, mediastinum, pleura, or intrathoracic lymph nodes, 5.34%); thoracoabdominal (22.93%); and “other” (2.62%). Data regarding the surgical approach or the types of CT regimens, postoperative complications, and metastasis arising after 6 months were not available in the registry databases. No data were available on the treatment of metastatic sites. We limited the study to describing the first-line management of patients with stage IV CRC at the time of diagnosis.

Socioeconomic status was defined using the European Deprivation Index (EDI), which is an ecological and composite indicator included in the census of the European Union’s statistics on income and living conditions. We compared quintiles 1 and 2 to quintiles 3, 4, and 5. For all cases, patient addresses were geo-localized using the geographic information system (ArcGIS 10.2) and assigned to a “Ilots Regroupés pour L’Information Statistique” (IRIS), a geographic area defined by the “Institut National de la Statistique et des Études Économiques” (INSEE). IRIS is the smallest geographical unit in France for which census data are available.

### 2.3. Statistical Analysis

The endpoint of follow-up in our study was 30 June, 2018. Of the 3000 patients with CRC and SM at the end of the follow-up period, 89.80% (*N* = 2694) were deceased, and 9.13% (*N* = 274) were alive. Thirty-two patients (1.07%) were lost to follow-up. To evaluate the impact of the different treatments on survival, analyses were performed from the date of diagnosis to the date of last contact (main objective). We used the Kaplan–Meier method to obtain survival curves and a Cox proportional hazards model to evaluate the impact of treatment sequence (CT vs. PTR) on survival in Group 4. To evaluate the potential benefit of treatment sequence on survival (secondary objective), survival was defined as the period between the date of the last treatment received and the date of the last contact for the group who received CT first, as there were no deaths in this group.

Hazard ratios (HRs) were calculated, using semi-parametric Cox hazard models, to evaluate the effect of therapeutic sequence (CT first vs. PTR first) on survival for patients with SM who were eligible for CT and surgery. The proportional risk assumption was verified (Schoenfeld residue). Variables with a threshold of *p* ≤ 0.20 on univariable analysis (M0) were included in the multivariable model (M1). The variable of interest (first treatment) was forced in all models. A value of *p* = 0.05 was considered statistically significant. All statistical analyses were performed using the StataSE14 software program (stataCorp LLC, College Station, TX, USA).

### 2.4. Missing Data

For the first objective, there were no missing values for treatment received. For the second objective, four variables had missing data: CCI (*N* = 255; 8.50%), EDI quintile (*N* = 15; 0.50%), extension of metastatic disease (*N* = 79; 2.63%), and discussion at multidisciplinary consultation (*N* = 314; 10.47%). Moreover, missing data on comorbidities and multidisciplinary tumor boards depended on other variables (respectively, age, department, deprivation for comorbidities, and year of diagnosis for multidisciplinary tumor boards).

Therefore, the missingness mechanism of these variables was probably not missing “completely at random”. Consequently, multiple imputation by chained equations (MICE) was performed to account for missing data (as recommended, the imputation model includes all variables available). Twenty imputations were considered sufficient, and 20 imputed datasets were produced using the multiple imputation command in STATASE14, according to Rubin’s rules. According to the guidelines on the presentation of results with missing data [11], results from the analysis of complete cases are presented in Table S1. After multiple imputations, HRs were calculated using Cox proportional hazard models to evaluate the effect of the first treatment performed (PTR vs. CT), after adjusting for confounding variables, among the 1159 patients who received both treatments (PTR + CT).

## 3. Results

### 3.1. Overall Population

**Figure 2:**
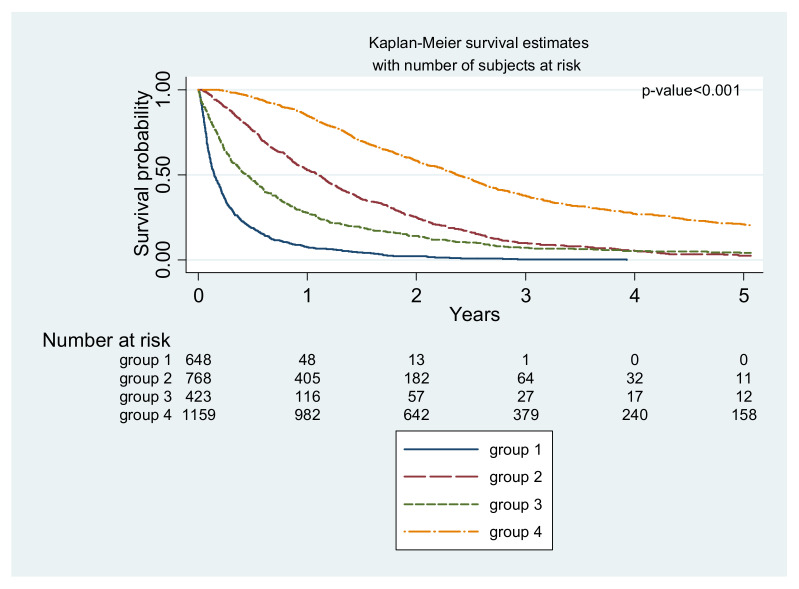
Overall survival of 3000 colorectal adenocarcinoma patients with synchronous metastasis, all by treatment (Kaplan-Meier curves).

The group distribution of the 3000 patients with CRC and SM included in our analysis was as follows: Group 1, 650 (21.67%) patients; Group 2, 768 (25.60%) patients; Group 3, 423 (14.10%) patients; and Group 4, 1159 (38.63%) patients. The population characteristics and survival for each treatment group are shown in Table 1. The crude survival rate was 51.80% at 1 year (95% confidence interval (CI), 50.00–53.60%) and 9.40% at 5 years (95% CI, 8.30–10.60%). The overall survival for each treatment group is shown in Figure 2, with the longest survival time observed in Group 4 (*p* < 0.001).

### 3.2. Group 1: Patients Receiving No Treatment or Only RT

This group included a higher proportion of females than males (53.54%), with the majority of patients being ≥80 years of age (62.15%) and with a CCI score > 2 (54.92%). The primary lesion was localized to the rectum in 24.46% of cases, with metastatic thoracoabdominal disease identified in 27.69%; 99.77% had uncomplicated CRC at the time of diagnosis. Almost a quarter of cases (25.08%) did not receive multidisciplinary consultation. The 1-year survival rate was 7.41% (95% CI, 5.60–9.60%); the 5-year survival rate was not available, due to the low number of patients who completed the 5-year follow-up.

For patients who received no treatment (*N* = 615) or RT alone (*N* = 35) the 1-year survival rate was significantly lower for colonic cancer (6.33%; 95% CI, 4.40–8.72) compared to rectal cancer (10.76; 95% CI, 6.54–16.16) (*p* = 0.013). The 5-year survival rate was not available, due to the low number of patients who completed the 5-year follow-up (Figure S1).

### 3.3. Group 2: Patients Receiving CT (+/− RT)

This group had a higher proportion of men (59.90%), with 15.10% of patients being >80 years of age, and 30.21% having a CCI score > 2. Almost two-thirds (61.46%) of cases were primary lesions of colonic origin, with metastatic intra-abdominal disease identified in 61.98%; 93.49% had uncomplicated CRC at the time of diagnosis. The majority of the cases (92.58%) were included in multidisciplinary consultation. The 1-year survival was 52.83% (95% CI, 49.23–56.29%), with a 5-year survival rate of 2.42% (95% CI, 1.37–3.96%).

For patients who received only CT (*N* = 604) or CT+RT (*N* = 164), the 1-year survival rate was significantly lower for colonic cancer (4.75%; 95% CI: 4.23–5.19) compared to rectal cancer (6.14; 95% CI, 5.57–6.67) (*p* = 0.03). The 5-year survival rate for colonic and rectal forms of CRC was 2.66% (95% CI, 1.35–4.72) and 1.85% (95% CI, 0.6–5.0%), respectively (Figure S1).

### 3.4. Group 3: Patients Receiving PTR (+/− RT)

Group 3 included a higher proportion of men than women (53.54%), with the majority of patients (62.15%) being ≥80 years of age, with a CCI score > 2 (54.92%). Nine out of ten cases (90.07%) were diagnosed with a primary lesion of colonic origin, with metastatic intra-abdominal disease identified in 76.12% of cases; 70.92% of cases had uncomplicated CRC at the time of diagnosis. Approximately 15% of cases (14.42%) did not receive multidisciplinary consultation. The 1-year survival was 27.55% (95% CI, 23.37–31.87%), with a 5-year survival rate of 4.12% (95% CI, 2.41–6.51%).

For patients who received only PTR or PTR + RT, there was no significant difference in survival between patients with colonic compared to rectal lesions (Figure S1).

### 3.5. Group 4: Patients Receiving PTR + CT (+/− RT)

This group had a higher proportion of men than women (58.41%), with a mean age of 66 (range, 22–88) years and a CCI score ≤ 2 (91.89%). The primary tumor was localized in the colon in the majority of cases (80.85%), with a unique location of SM in 70.66% of cases, which was principally intra-abdominal (74.20%). The main features of management included multidisciplinary consultation (93.10%), with colectomy performed in 73.34% (*N* = 850) of case and proctectomies in 25.71% (*N* = 298). Among the 1159 patients in this group, PTR was performed on an emergency basis in 171 (14.75%). The 1-year survival rate was 84.83% (95% CI, 82.53–86.68%), with a 5-year survival of 21.01% (95% CI, 18.50–23.65%). The majority of patients in this group were first treated with PTR (77.57%) rather than CT (22.43%). PTR as the first treatment was more frequently performed for colonic than rectal tumors (87.48% vs. 36.04%, respectively, *p* = 0.001), with CT being the first treatment in the majority of cases of colonic tumors (63.96%) compared to rectal tumors (12.59%).

In a univariable analysis (Table 2), the survival rate was lower for patients >80 years of age than for those <60 years of age: Cox regression HR, 2.37 (95% CI, 1.86–3.00). Patients diagnosed in the most recent period (2013–2016) had better survival than those diagnosed in the previous period (2006–2008): HR, 0.81 (95% CI, 0.68–0.96). Patients with a higher CCI (score > 2) had a higher risk of mortality than those with a low CCI (score ≤ 2): HR, 1.56 (95% CI, 1.32–1.85). Finally, a thoracoabdominal localization of SM was associated with worse survival (HR, 1.56; 95% CI, 1.32–1.84). The first treatment received (PTR or CT) was not associated with better survival (HR, 0.95; 95% CI, 0.80–1.11; *p* = 0.50).

In a multivariable regression model (M1) adjusting for age, year of diagnosis, CCI, and the localization of SM, the sequence of treatment was not significantly associated with survival (HR, 1.05; 95% CI, 0.88–1.84; *p* = 0.55).

A subgroup analysis according to the year at diagnosis was performed. After adjusting for age, sex, CCI, and metastatic characteristics, there was no significant advantage in survival for CT first, regardless of the year of diagnosis (HRa _2006–2008_ = 0.81 (0.54–1.23); HRa _2009–2012_ = 1.09 (0.79–1.50); HRa _2013–2016_ = 1.19 (0.82–1.74) (data not shown)). After adjusting for age, sex, CCI, metastatic characteristics, there is no difference according to metastatic site (HRa _intra-abdominal_ = 1.03 (0.80–1.32); HRa _intrathoracic_ = 1.82 (0.72–4.60); HRa _thoracoabdominal_ = 0.73 (0.46–1.14) (data not shown)). We also analyzed patients with CCR and those who had received RT for rectal cancer separately. Treatment with RT did not have a significant effect on overall survival in patients with stage IV rectal cancer who received PTR and CT (HR _radio yes/radio no_ = 0.74 (0.38–1.44)).

For the 1159 patients who received a combination of two treatments (PTR + CT, *N* = 1101; PTR + CT + RT, *N* = 58) the 1-year survival rate for colonic and rectal lesions was 2.78% (95% CI, 2.32–3.22) and 2.62% (95% CI, 1.41–4.00), respectively. The 5-year survival rate for colonic lesions was 2.62% (95% CI, 1.41–4.00). The 5-year survival rate for rectal forms was not available because of the small number of patients who completed the 5-year follow-up (Figure S1).

## 4. Discussion

It was not surprising that the prognosis of patients diagnosed with CRC and SM was markedly poor. However, for a highly selected population of patients who received combination treatment (PTR + CT), representing 38% of our study population, the median 5-year survival increased by up to 21%, regardless of the treatment sequence (CT vs. PTR). Unfortunately, outside of this therapeutic option, the 5-year median survival for patients with CRC and SM was <5%.

These results illustrate the heterogeneity of practices and the very high percentage of first colorectal resections in spite of SM. Our findings illustrated the difficulty of applying the recommended clinical practice guidelines and the lack of standardization of management for patients with CRC and SM. Moreover, the high percentage of first colorectal resections is in line with the results of a published meta-analysis on this topic, which included 26 studies [12].

The majority of studies have focused on therapeutic strategies for CRC and synchronous liver metastases, which is the most frequent site of SM disease [13,14]. However, patients participating in these studies, whether randomized or from centers with expertise in both oncology and surgery, are generally selected to receive the various treatments and, thus, have a better health status than the general population with CRC and SM. Therefore, there is a need for studies that take into account all patients with CRC and SM.

In our study, different treatment strategies were used, including no specific oncological treatment (21.60%), CT with or without radiotherapy RT (25.5%), and PTR with or without RT (14%). Only 38.5% of our patient sample was, however, treated with PTR + CT. On an intention-to-treat basis, patients with a primary stoma were included in the PTR + CT group only if colorectal oncologic resection was performed at the end of the treatment sequence. Consequently, patients with only bypass (3.3%) or stoma (18%) and without tumor resection were included in Group 1. At 1 year, the crude survival ranged from 7.41% (no treatment or only PTR) to 84.83% (PTR + CT). However, this therapeutic strategy could only be applied to about 40% of patients.

These results are broadly consistent with those published by Xu et al. [13], using a hospital-based cancer registry (NCDB). Among the 31,310 patients with stage IV colon cancer, 22% of the patients underwent PTR with postoperative CT, 37.5% received only CT, 11.9% underwent only PTR, and 28.6% received no treatment. According to the Surveillance, Epidemiology, and End Results (SEER) database of the National Cancer Institute, 1975–2016, the survival rates for different treatment strategies were as follows: no treatment, 12.6%; surgery alone, 12.4%; chemotherapy alone, 27.3%, and surgery combined with chemotherapy, 29.9% [13].

In a case–control study based on a national registry of patients with stage IV CRC and SM, Afshari et al. [15] evaluated patient, tumor, and treatment-related prognostic factors associated with survival. No patients were treated with a liver-first strategy. All patients with resected synchronous stage IV CRC who survived >5 years were included as cases. The control group consisted of corresponding patients who lived <5 years. For liver resection and lung resection, no significant differences were found between the groups. Multivariable analysis showed that the most important predictor of achieving a lifespan >5 years was metastasectomy (HR, 8.26; 95% CI, 3.98–16.95). Multivariable analysis confirmed that stage III-IV CRC at the time of diagnosis (HR, 3.70; 95% CI, 2.89–4.99; *p* < 0.0001) and the presence of colonic origin (HR, 1.30; 95% CI, 1.01–1.69; *p* = 0.04) were the main prognostic factors for survival [15].

In a first phase III randomized controlled trial, Kanemitsu et al. [7] evaluated the survival benefits of combining PTR with CT for patients with asymptomatic CRC (colon; *N* = 153 or upper rectal adenocarcinoma; *N* = 12) and up to three unresectable metastases confined to liver, lung, distant lymph nodes, or peritoneum. The trial was conducted over a 7-year period at 38 Japanese healthcare and medical centers. In total, 84 patients were assigned to the CT-only group and 81 patients to the PTR + CT group. At the first interim analysis, the trial was stopped prematurely because of futility. With a median follow-up of 22.0 months, the median survival was 25.9 months (95% CI, 19.9–31.5) in the PTR + CT group and 26.7 (95% CI, 21.9–32.5) in the CT alone group (HR, 1.10; 95% CI, 0.76–1.59; *p* = 0.69). This study highlights the importance of not offering RTP to all patients with stage IV CRC. There is a real benefit in terms of overall survival only in a well-selected population. [7]

In our study, we evaluated survival outcomes for each therapeutic strategy. Overall survival was significantly improved in patients undergoing PTR + CT. At 1 year, the crude survival ranged from 7.41% (no treatment or RT only) to 84.83% (PTR + CT). Our results are in line with recent reports on survival based on cancer registry data [13,14]

In their systematic review and network meta-analysis, Ghiasloo et al. [16] concluded that the surgical management of patients with CRC and SM should be individualized owing to the absence of evidence of a survival advantage according to the treatment strategy.

This study has some limitations. Despite the use of high-resolution-based studies, some variables need further investigation.

The absence of information concerning the patient, the intent of treatment decided during the multidisciplinary consultation, the surgical procedure, the assessment of resectability (i.e., treatment of metastatic site, by either surgery or chemotherapy intraperitoneal hyperthermic), the type and round of chemotherapy, and postoperative outcomes limit the reliability of the results. In addition, the prevalence of medical files not presented at multidisciplinary consensus meetings was 7.5–25% in Group 1. This result can be explained by several factors, including the long inclusion period of the patients, the involvement of low- and high-volume centers, and palliative intent in Group 1. In France, digestive cancer registries collect information on medical files thanks to specialized trained technicians. No medical information is automatically provided by the healthcare sector to cancer registries. It is challenging to collect data on disease stage, the healthcare setting where treatment was administered, and the completed treatment. This is the main additional information provided by specialized registries compared to nonspecialized cancer registries. Other variables such as molecular biology and the use of monoclonal antibodies or radiotherapy schemes were missing in medical files for the vast majority of patients. Consequently, such variables could not be included in our study and, thus, these constitute a limitation of our study.

The information on metachronous metastasis could not be included in our analysis.

Some authors have shown that SM at the time of CRC diagnosis is associated with poorer survival than metachronic metastatic disease. In contrast, Engstrand et al. [17] reported that, for patients with CRC who reside in the cities of Stockholm and Gotland, Sweden, identification of synchronous or metachronic liver metastases had no prognostic value on survival from CRC [17]

It is important to also note the strengths of our study. Despite the aforementioned lacking variables, unlike hospital-based series, population-based registries ensure exhaustiveness of cases and heterogeneity of management in the two geographical areas, regardless of the level of specialization of the therapeutic management. Moreover, the long period of inclusion allowed us to highlight the importance of therapeutic guidelines. From a statistical point of view, the use of the most updated method for dealing with missing data was also a strength.

## 5. Conclusions

In summary, our results indicate that CT + PTR is the cornerstone of the oncological management of CRC with SM, with survival being negatively impacted by other treatment strategies. Moreover, our data identified that the sequence of treatment, CT vs. PTR first, resulted in comparable oncologic prognosis. The initial diagnostic assessment is crucial to ensure access to combined treatment for a large number of patients.

Almost 40% of patients could benefit from this therapeutic combined strategy. In the future, the inclusion of more patients eligible for this combined strategy could dramatically improve prognosis. This strategy requires the early identification of these heterogeneous patients by means of multidisciplinary consultation meetings in specialized centers, including the use of modern techniques such as telemedicine, screening access, and symptom awareness. Ideally, a personalized therapeutic approach would take into account the individual molecular characteristics of the tumor and evaluate the response to the proposed treatment.

## Figures and Tables

**Table 1:** Population characteristics and survival for each treatment group (*N* = 3000).

	Group 1		Group 2		Group 3		Group 4		Total	
	No Treatment or Only RT		CT [+/− RT]		PTR [+/− RT		PTR + CT [+/− RT]			
	*N* = 650	%	*N* = 768	%	*N* = 423	%	*N* = 1159	%	*N* = 3000	%
**Sex**										
Men	302	46.46	460	59.90	216	51.06	677	58.41	1655	55.17
Women	348	53.54	308	40.10	207	48.94	482	41.59	1345	44.83
**Age group**										
<60	19	2.92	194	25.26	18	4.26	327	28.21	558	18.60
60–69	87	13.38	248	32.29	39	9.22	367	31.67	741	24.70
70–79	140	21.54	210	27.34	98	23.17	359	30.97	807	26.90
>=80	404	62.15	116	15.10	268	63.36	106	9.15	894	29.80
**Year of diagnosis**										
2006–2008	123	18.92	162	21.09	124	29.31	322	27.78	731	24.37
2009–2012	247	38.00	268	34.90	163	38.53	414	35.72	1092	36.23
2013–2016	280	43.08	338	44.01	136	32.15	423	36.50	1177	39.05
**Department**										
Calvados	252	38.77	332	43.23	190	44.92	441	38.05	1215	40.50
Finistere	398	61.23	436	56.77	233	55.08	718	61.95	1785	59.50
**Charlson index**										
0	90	13.85	206	26.82	83	19.62	306	26.40	685	22.83
1 or 2	135	20.77	256	33.33	90	21.28	369	31.84	850	28.33
>2	357	54.92	232	30.21	231	54.61	390	33.65	1210	40.33
missing	68	10.46	74	9.64	19	4.49	94	8.11	255	8.50
**Localisation of primary tumor** ^a^										
colon	491	75.54	472	61.46	381	90.07	937	80.85	2281	76.03
rectum	159	24.46	296	38.54	42	9.93	222	19.15	719	23.97
**Number of metastatic disease**										
multiple	270	41.54	334	43.49	125	29.55	340	29.34	1069	35.63
unique	380	58.46	434	56.51	298	70.45	819	70.66	1931	64.37
**Metastatic disease**										
intra-abdominal	415	63.85	476	61.98	322	76.12	860	74.20	2073	69.10
intra-thoracic	36	5.54	35	4.56	26	6.15	63	5.44	160	5.33
thoraco-abdominal	180	27.69	234	30.47	60	14.18	214	18.46	688	22.93
others ^b^	11	1.69	12	1.56	3	0.71	2	0.17	79	2.63
**Multidisciplinary consultation**										
yes	326	50.15	711	92.58	299	70.69	1079	93.10	2415	80.50
no	163	25.08	25	3.26	61	14.42	22	1.90	271	9.03
missing	161	24.77	32	4.17	63	14.89	58	5.00	314	10.47
**Type of surgery**										
colectomy	0	0.00	0	0.00	360	85.11	850	73.34	1210	40.33
protectomy	0	0.00	0	0.00	56	13.24	298	25.71	354	11.80
internal by-pass	21	3.23	13	1.69	0	0.00	0	0.00	34	1.13
permanent stoma	117	18.00	155	20.18	0	0.00	0	0.00	272	9.07
exploratory laparotomy	32	4.92	80	10.42	0	0.00	0	0.00	112	3.73
metastasectomy	1	0.15	6	0.78	0	0.00	0	0.00	7	0.23
others resections	0	0.00	0	0.00	4	0.95	11	0.95	15	0.50
no surgery	479	73.69	514	66.93	0	0.00	0	0.00	993	33.10
unknown	0	0.00	0	0.00	3	0.71	0	0.00	3	0.10
**Complicated primary tumor**										
yes	97	14.92	69	8.98	132	31.21	200	17.26	498	13.90
no	553	85.08	699	91.02	291	68.79	959	82.74	2502	86.10
**EDI quintile group**										
1-2	298	45.85	369	48.05	163	38.53	573	49.44	1403	46.77
3-4-5	346	53.23	395	51.43	259	61.23	582	50.22	1582	52.73
missing	6	0.92	4	0.52	1	0.24	4	0.35	15	0.50
**Survival (95% CI)**										
**1-year**	7.41 (5.60 to 9.60)		52.83 (49.23 to 56.29)		27.55 (23.37 to 31.87)		84.83 (82.53 to 86.68)		51.80 (50.00 to 53.60)	
**5-year**	-^c^		2.42 (1.37 to 3.96)		4.12 (2.41 to 6.51)		21.01 (18.50 to 23.65)		9.40 (8.30 to 10.60)	

^a^ rectosigmoid were included in the colic localisation (*N* = 711); ^b^ 51 unknown and 28 others; ^c^ no results for no treatment because of number of deaths (*N* = 2).

**Table 2:** Hazard ratios with 95% confidence limits from Cox proportional hazards imputed models (*N* = 1159) (survival between the date of last treatment and end point).

	M0			M1		
	HR	95% CI	*p* Value	HR	95% CI	*p* Value
**Treatment first**						
PTR	1	Ref	0.50	1	Ref	0.55
CT	0.95	0.80 to 1.11		1.05	0.88 to 1.24	
Sex						
Men	1	Ref	0.36			
Women	1.07	0.93 to 1.22				
**Age (years)**						
<60	1	Ref	**0.001**	1	Ref	**0.001**
60–69	1.12	0.93 to 1.34		1.09	0.91 to 1.32	
70–79	1.66	1.39 to 1.98		1.47	1.21 to 1.79	
>=80	2.37	1.86 to 3.00		2.05	1.58 to 2.66	
**Year of diagnosis**						
2006–2008	1	Ref	**0.03**	1	Ref	0.09
2009–2012	0.85	0.73 to 1.00		0.88	0.75 to 1.03	
2013–2016	0.81	0.68 to 0.96		0.83	0.70 to 0.99	
**Departement**						
Calvados	1	Ref	0.28			
Finistere	1.08	0.94 to 1.24				
**Charlson comorbidity index**						
0	1	Ref	**0.001**	1	Ref	**0.01**
1 ou 2	0.99	0.83 to 1.18		1.03	0.86 to 1.15	
>2	1.56	1.32 to 1.85		1.30	1.08 to 1.57	
**Localisation of primary tumor**						
colic	1	Ref	0.48			
rectal	0.94	0.79 to 1.11				
**Localisation of metastatic disease**						
intra-abdominal	1	Ref	**0.001**	1	Ref	**0.001**
intra-thoracic	0.99	0.73 to 1.33		0.90	0.67 to 1.22	
thoraco-abdominal	1.56	1.32 to 1.84		1.53	1.30 to 1.80	
**Number of metastatic site**						
unique	1	Ref	**0.01**			
multiple	0.51	0.45 to 0.59				
**EDI quintile**						
1, 2	1	Ref	0.92			
3, 4, 5	1.00	0.88 to 1.15				

## Data Availability

Data and code are available upon reasonable request.

## Supplementary Materials

The following supporting information can be downloaded at: https://www.mdpi.com/article/10.3390/cancers14071777/s1, Figure S1: Kaplan-Meier Curves for each treatment groups according to sub-localisation; Table S1: Hazard ratio with 95% confidence limits from Cox proportional hazards models in the group 4 (*N* = 1159) on non imputed data.
