# Supplementary material for: Care Management and Survival of Patients Diagnosed with Synchronous Metastatic Colorectal Cancer: A High-Resolution Population-Based Study in Two French Areas
Source: Cancers (Basel). 2022 Mar 31;14(7):1777. doi: 10.3390/cancers14071777 (PMC8997002; doi:10.3390/cancers14071777)
Supplement: Supplementary file 1 [file cancers-14-01777-s001.zip › cancers-1619696-supplementary.pdf]

Table S1. Hazard ratio with 95% confidence limits from Cox proportional hazards models in the group 4 (*n* = 1159) on non imputed data.

		M0					M1		
**Characteristic**		**N**	**Deaths (N)**	**HR**	**95% CI**	**P value**	**HR**	**95% CI**	**P value**
**Treatment first**									
	PTR	899	674	1	Ref	0.50	1	Ref	0.61
	CT	260	173	0.95	0.80 to 1.11		1.05	0.87 to 1.26	
**Sex**									
	Men	677	577	1	Ref	0.36			
	Women	482	424	1.07	0.93 to 1.22				
**Age (years)**									
	<60	327	268	1	Ref	**<0.001**	1	Ref	**<0.001**
	60-69	367	305	1.12	0.93 to 1.34		1.08	0.89 to 1.31	
	70-79	359	326	1.66	1.39 to 1.98		1.41	1.13 to 1.75	
	>= 80	106	102	2.37	1.86 to 3.00		2.06	1.56 to 2.70	
**Year of diagnosis**									
	2006-2008	322	269	1	Ref	**0.04**	1	Ref	0.11
	2009-2012	414	324	0.85	0.73 to 1.00		0.84	0.71 to 1.00	
	2013-2016	423	408	0.81	0.68 to 0.96		0.85	0.71 to 1.02	
**Departement**									
	Calvados	441	371	1	Ref	0.27			
	Finistere	718	630	1.08	0.94 to 1.24				

**Charlson comorbidity index**								
0	306	253	1	Ref	**<0.001**	1	Ref	**0.01**
1 ou 2	369	300	0.99	0.83 to 1.19		1.02	0.84 to 1.23	
>2	390	364	1.59	1.34 to 1.90		1.32	1.08 to 1.60	
**Localisation of primary tumor**								
colic	937	811	1	Ref	0.47			
rectal	222	190	0.94	0.79 to 1.11				
**Localisation of metastatic disease**								
intra-abdominal	860	723	1	Ref	**<0.001**	1	Ref	**<0.001**
intra-thoracic	63	55	0.88	0.73 to 1.32		0.92	0.67 to 1.25	
thoraco-abdominal	214	204	1.57	1.33 to 1.85		1.58	1.33 to 1.88	
**Number of metastatic site**								
unique	819	677	1	Ref	**<0.001**			
muliple	340	324	1.95	1.69 to 2.24				
**EDI quintile**								
1.2	573	501	1	Ref	0.91			
3.4.5	582	496	1.01	0.88 to 1.15				

Groupe 1 : No treatment or only RT

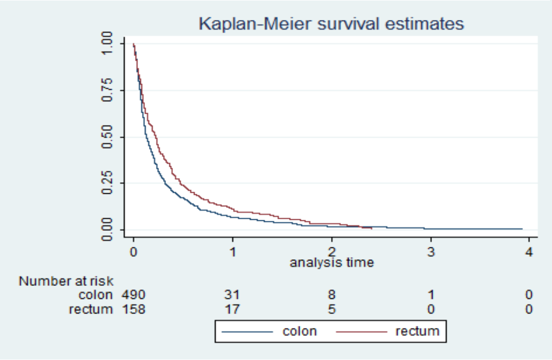

Groupe 2 : only CT or CT +RT

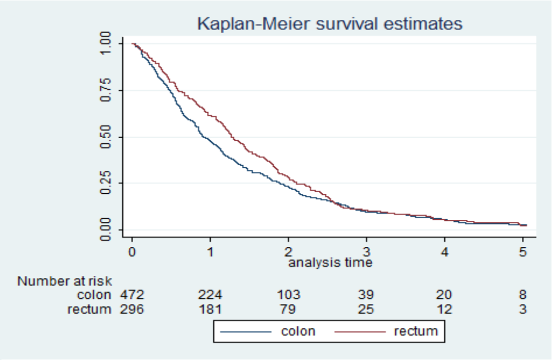

Groupe 3 : Only PTR or PTR + RT

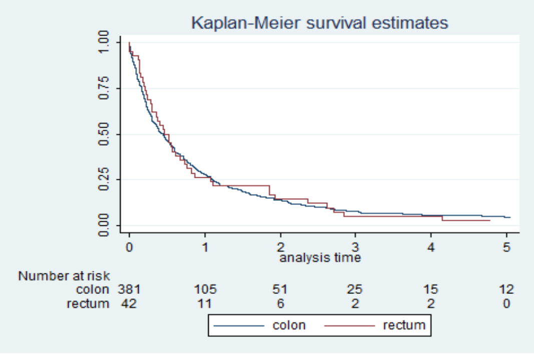

Groupe 4 : PTR + CT or PTR+ CT+ RT

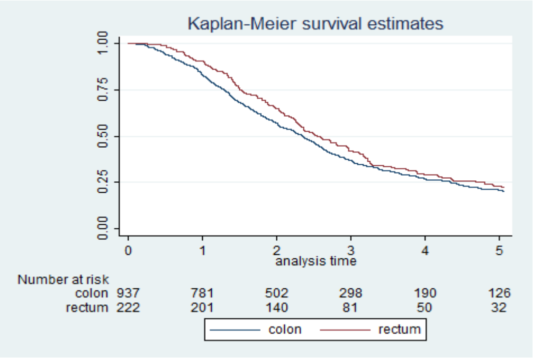

Figure S1. Kaplan-Meier Curves for each treatment groups according to sub-localisation (Colon Vs Rectum).
